# Prognostic importance of DNA from human papillomavirus in patients with oral squamous cell carcinoma

**DOI:** 10.4317/medoral.25092

**Published:** 2022-02-20

**Authors:** Elizabeth Pérez-Islas, Alejandro García-Carrancá, Enrique Acosta-Gio, Nancy Reynoso-Noverón, Héctor A Maldonado-Martínez, Miriam Guido-Jiménez, Nora Sobrevilla-Moreno, Martín Granados-García, Wendy B Pérez-Báez, Diana Vilar-Compte

**Affiliations:** 1 Posgrado en Ciencias Médicas, Odontológicas y de la Salud, Facultad de Medicina, National Autonomous University of Mexico, Mexico City, Mexico; 2 Hospital Epidemiology, Department of Infectious Diseases, Instituto Nacional de Cancerología, Mexico City, Mexico; 3 Virus and Cancer Laboratory, Unit of Biomedical Research on Cancer, Instituto de Investigaciones Biomédicas, National Autonomous University of Mexico and Instituto Nacional de Cancerología, Mexico City, Mexico; 4 Microbiology Laboratory, Division of Postgraduate Studies and Research, National Autonomous University of Mexico, Mexico City, Mexico; 5 Epidemiology Unit, Instituto Nacional de Cancerología, Mexico City, Mexico; 6 Department of Surgical Pathology, Instituto Nacional de Cancerología, Mexico City, Mexico; 7 Department of Medical Oncology, Instituto Nacional de Cancerología, Mexico City, Mexico; 8 Head and Neck Surgery Department, Instituto Nacional de Cancerología, Mexico City, Mexico; 9 Department of Molecular Biology, Instituto Nacional de Cancerología, Mexico City, Mexico

## Abstract

**Background:**

Survival of patients with oral squamous cell carcinoma (OSCC) is generally low, with the likelihood of locoregional recurrence or disease progression (LR/DP). Knowledge of prognostic factors for survival is key to achieving an understanding and increased survival. The present study aimed to identify prognostic factors for patients with OSCC, especially the presence of DNA from human papillomavirus (HPV).

**Material and Methods:**

Retrospective cohort study including 119 patients with OSCC treated at the National Cancer Institute in Mexico City (2009-2013). Clinical information was obtained from patient records including LR/DP. Formalin-fixed, paraffin-embedded tissues were obtained and used for detecting DNA from different types of HPV. Potential prognostic factors for Overall Survival (OS) were analyzed using the Cox proportional hazards model.

**Results:**

After model adjustment, factors associated with longer OS were a pre-treatment platelet count above 400,000/mm3 (HR=0.09, *p*=0.026) and response to primary treatment (HR=0.26, *p*=0.001). HPV DNA was present in 23 (19.3%) of the patients and importantly, type 16 found in 19 of them. Although survival of HPV-positive patients was longer, difference was not significant. However, among patients with LR/DP, HPV positivity was significantly associated with increased survival (HR=0.23, *p*=0.034). Importantly, survival was significantly different for HPV-positive patients with LR/DP > 6 months (HR=0.20, *p*=0.002), had higher absolute lymphocyte count at start of treatment (HR=0.50, *p*=0.028) or had local rescue treatment (HR=0.24, *p*=0.019).

**Conclusions:**

Although HPV positivity was not associated with a longer OS of OSCC patients, a better prognosis was significantly associated with HPV positivity and recurring or progressing disease, particularly with HPV type 16.

** Key words:**HPV, human papillomavirus, HPV-16, oral squamous cell carcinoma, oral cancer, survival, locoregional recurrence, disease progression.

## Introduction

In the last two decades, an increased incidence of oropharynx squamous cell carcinoma (OPSCC) and, more recently, oral squamous cell carcinoma (OSCC) has been observed (1). As a result, health systems need to allocate more resources to provide specialized care (2). This situation is aggravated by late diagnosis, with a low probability of response to treatment (3), increased likelihood of locoregional recurrence/disease progression (LR/DP), and lower overall survival (OS) (4). Specifically, in the OSCC, LR/DP occurs in up to 50% of patients (5,6).

The prognosis of patients with OSCC is associated with the clinical stage, the oncological treatment, and some clinical and pathologic characteristics. All of the above provide valuable information that guide treatment (7-9). It is well known that human papillomavirus (HPV) infection in OPSCC is associated with a better prognosis than that observed among carcinomas attribuTable to tobacco and alcohol; however, in OSCC, the importance of HPV infection is less clear, and the information is even contradictory (10,11).

A significant group of patients with OSCC will not be free of the disease or will recur after completing the planned treatment. If recurrence occurs, treatment is even more difficult because of the therapeutic limitations of the previous treatment, surgical sequelae, and the late toxicity of chemotherapy and radiotherapy (5).

Given the better oncologic outcomes in HPV-positive OPSCC patients, de-escalation of treatment has been proposed to reduce toxicity (12). However, the prognostic significance of HPV-positivity in OSCC tumors is less clear. Therefore, we performed a retrospective cohort study to identify prognostic factors in OSCC, focusing on the importance of HPV DNA among patients with LR/DP.

## Material and Methods

- Population and setting

Patients with a diagnosis of non-metastatic OSCC, including (C00.5, C02.3, C03.9, C04.9, C05.0, C06.0, and C06.2) of the International Classification of Diseases (ICD-10) were included. All patients were admitted at INCan (Mexico City, MEXICO) between February 2009 and December 2013. Data available until December 2016 was collected and analysis performed between August 2018 and December 2020.

- Variables and Outcomes

Relevant information was obtained from the patient's clinical records, specimens, and histopathological records, including age, sex, smoking, alcohol consumption, absolute lymphocyte count (103/mm3), and platelets count before treatment. The clinical stage was defined according to the American Joint Committee on Cancer (AJCC), 7th edition. Moreover, patients were grouped as an early disease (I and II), or advanced disease (III and IVa).

- Treatment

Patients were treated according to institutional protocols, which include surgery (S) as first choice, or initial radiotherapy (RT), for patients with early disease. Surgery plus radiotherapy (S+RT) or chemoradiotherapy (S+CRT) according to the risk of recurrence for patients with moderately advanced disease (resecTable), or induction chemotherapy followed by chemoradiotherapy (iCT+CRT), or exclusively concomitant chemoradiotherapy (CRT) for patients who were resecTable but refused surgery, or for those unresecTable.

In the case of LR/DP, rescue surgery with curative intent was considered, but if this was not feasible due to the extent of the disease or the patient's conditions, palliative treatment was provided.

- Response to treatment and follow-up

The follow-up time of the cohort was seven years. Response to treatment was evaluated from six to eight weeks after completion of surgery or radiotherapy. Patients that received concomitant CRT were evaluated twelve weeks after completion of treatment. The evaluation was done with magnetic resonance imaging (MRI), or PET-CT with 18-FDG and under the RECIST criteria version 1.1. Patients were grouped for treatment response as responders (complete or partial response) or non-responders (disease progression).

Patients with therapeutic failure were those with LR/DP. During follow-up, patients with LR/DP included those who were never disease-free or recurred. Time to LR/DP was grouped into > six months or ≤ six months after completion of planned treatment until the end of the cohort.

OS was calculated from the date of diagnosis to death, or to the date of the last follow-up visit.

- Detection of HPV DNA and typing

For DNA extraction, ten μm thick sections were trimmed from the formalin-fixed paraffin tissue (FFPE) selected from each patient and corresponding biopsy at the time of diagnosis or surgery and collected in a microtube. DNA was isolated from this tissue using the QIAamp® DNA FFPE Tissue Kit (QIAGEN, Hilden, Germany) following the manufacturer´s protocol (13).

DNA sample concentration was estimated by spectrophotometry on a Nano Photometer 1000V3 (Thermo Fisher Scientific, Massachusetts, USA). HPV DNA was detected using INNO-LiPA HPV Genotyping Extra II Amp kit (INNO-LiPA) (Innogenetics, Belgium) (14), with 500 ng of DNA using the Auto-LiPA 48. The positive control was the kit’s HPV-6 DNA. Wistar rat kidney DNA served as the negative control to ensure that there was no cross contamination.

- Statistical analyses

To summarize the results, the HPV status, clinical and pathologic characteristics were described using means and standard deviations or medians, and interquartile ranges as appropriate. Categorical variables were described by absolute numbers and proportions. For the bivariate analysis, the chi-square test or Fisher exact test were used for categorical variables and Student t-test or Mann-Whitney U test for continuous variables, as appropriate.

Potential prognostic factors in patients with OSCC were analyzed by bivariate analysis to develop an OS model; the association was estimated by Cox proportional hazard model analysis, using a hazard ratio (HR) and 95% confidence intervals (CI). Proportionality over time and collinearity between covariates were calculated.

Cox’s model for regression of proportional risk was applied to evaluate the variables’ independent prognostic value for OS. A sub-analysis was performed to identify prognostic factors among patients with LR/DP, using a stratified model for OS. Unadjusted survival curves were obtained using the Kaplan-Meier method and compared with the Wilcoxon test. All data analyses were performed with Stata v.12 (StataCorp, College Station, Texas). A critical value of *p*<0.05 was considered significant.

## Results

- Patients

**Figure 1:**
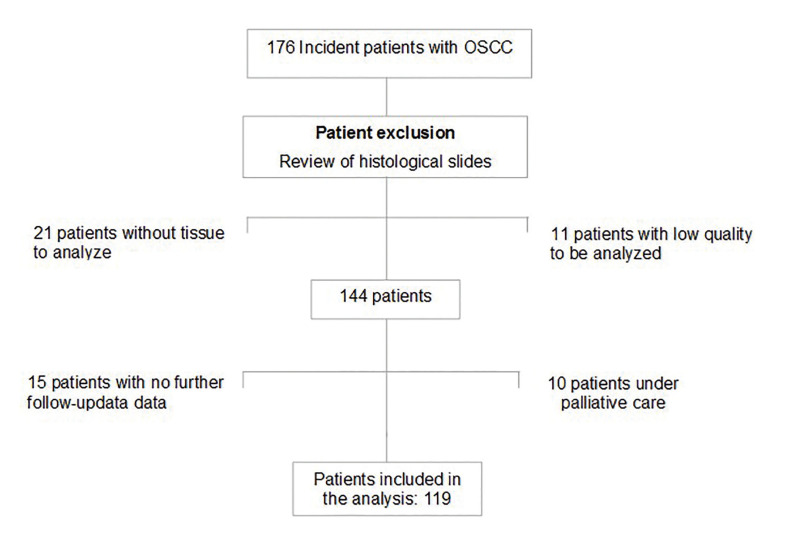
Diagram of patients with OSCC.

Of 176 incident cases with OSCC, 119 patients (51 males and 68 females) had complete information and paraffine blocks available for analysis. (Fig. 1). The mean age at diagnosis was 61.4 ± 14.2 years (25 to 94 years). Fifty-four (45.4%) patients were in stages I or II, and 65 (54.6%) in stages III or IVa, respectively. Demographic, clinical, and laboratory variables are shown in Table 1.

**Table 1:**
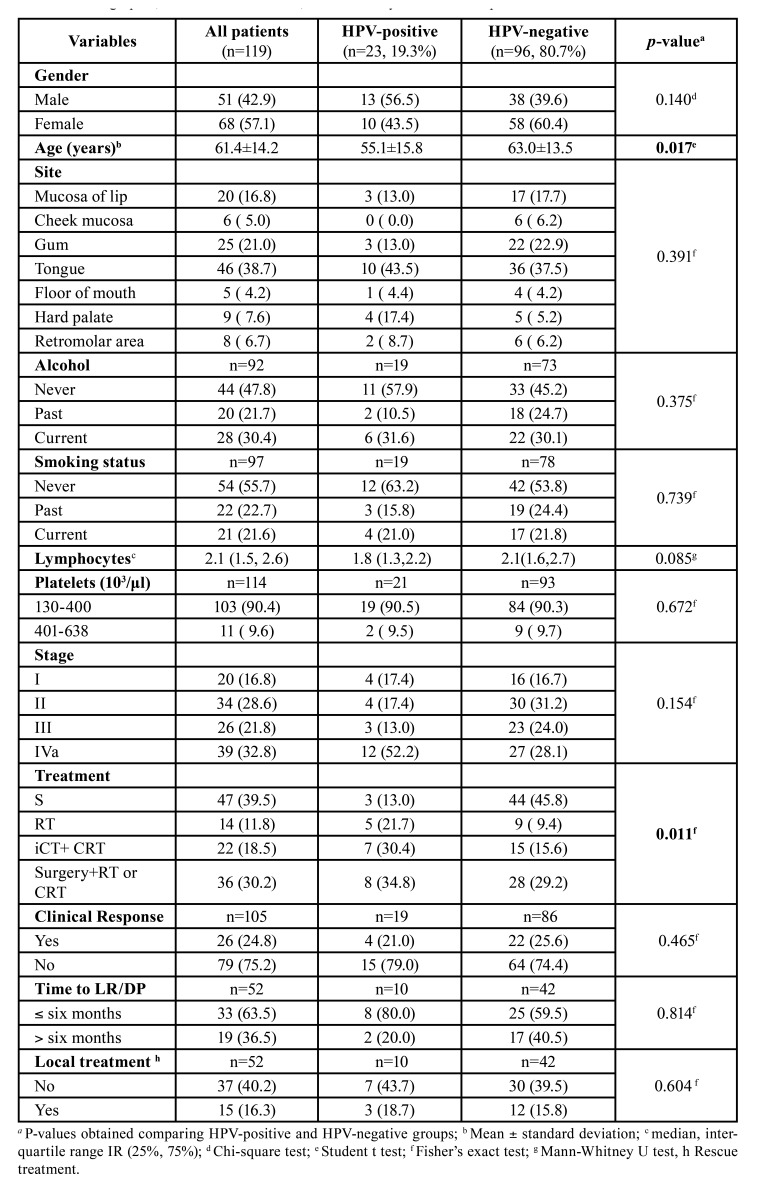
Demographic, clinical characteristics, and laboratory tests of OSCC patients.

- Frequency of HPV DNA

Of 119 tumor samples, 23 were positive for HPV DNA (19.3%). While the majority (n=22) contained at least one high-risk type (18.5%), only one sample was positive for low-risk type (HPV-44, 0.8%). The HPV types detected were: 6, 11, 16, 18, 44, 52, 58 and 66.

As expected, the most prevalent type was HPV-16 (16%), found in 19 samples and the only high-risk type in 14 of them. Only two other tumors were positive for one high-risk type (HPV-18 and 58). Among six tumors with more than one viral type, HPV-16 was present in five of them: HPV-6; 18; 66; 18 and 66; 11, 52 and 66, while HPV-18 presented coinfection with HPV-11. In the ten patients with LR/DP, nine (90%) had HR-HPV DNA.

HPV positivity was associated with younger age (*p*=0.017). Thirteen (56.5%) of 23 HPV-positive samples were from men (*p*=0.140).

- Treatment, and clinical course

Among the 54 patients with early disease (stage I and II), 29 (24.4%) were treated with surgery exclusively, 8 (6. 7%) with radiotherapy (RT) exclusively, and 17 (14.3%) required combined treatment: 14 were with surgery plus radiotherapy, or chemoradiotherapy (S+RT or CRT) indicated by positive margins not amenable to extension or some metastatic node with capsular rupture, and three, were treated with induction chemotherapy followed by concomitant chemoradiotherapy (iCT+CRT), because they declined surgery because of unaccepTable sequelae.

Among the 65 patients with advanced clinical stages (III and IVa), 18 (15.1%) were treated with surgery alone and 6 (5%), only with RT, as they were not suiTable for multimodal treatment; 41 (34.4%) received combined treatment, of these, 19 with iCT+ CRT, and 22 with S+ RT or CRT.

Out of 119 patients analyzed, 52 (43.7%) developed LR/DP, and of these, ten were HPV-positive.

- Survival analysis

The median follow-up was 2.17 years with a range of 1.5 months to 7 years, 35 (29.4%) patients died of the disease. The median follow-up to LR/DP was 1.9 years with a range between 2 months to 5.6 years.

**Figure 2:**
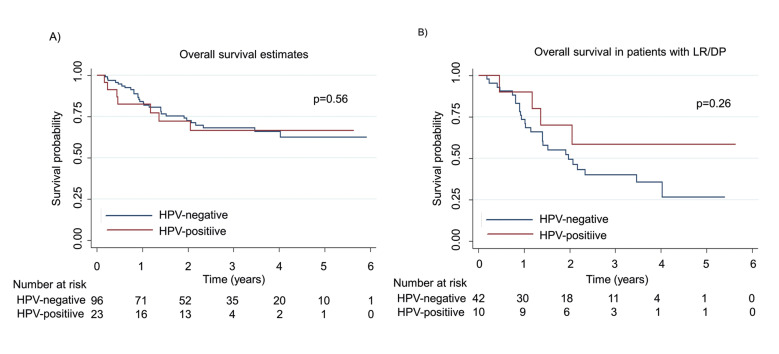
A) Overall survival illustrated with Kaplan-Meier curve stratified by HPV status. The median OS was 2.05 years for HPV-positive and 2.23 years for HPV-negative patients (*p*=0.56); B) Overall survival in patients with LR/DP and stratified by HPV status illustrated with Kaplan-Meier curve. HPV-positive patients had a median of 2.13 years and 1.47 years for HPV-negative patients (*p*=0.26).

There were no differences in follow-up between HPV-positive and HPV-negative patients (2.05 and 2.23 years respectively, *p*=0.56). However, when analyzing OS among patients who developed LR/DP, HPV-positive patients had a median survival of 2.13 years, compared with only 1.47 years among HPV-negative patients (*p*=0.26) (Fig. 2).

In the model adjusted for OS, advanced clinical stage (HR=2.75, 95% CI: 1.20-6.27, *p*=0.016), primary treatment with RT (HR=4.52, 95% CI: 1.18-17.3, *p*=0.027), or iCT+RT or CRT (HR=4.04, 95% CI: 1.40-11.6, *p*=0.010), were independent prognostic factors associated with poorer survival, while elevated platelet count (HR=0.09, 95% CI: 0.01-0.75, *p*=0.026) and response to primary treatment (HR=0.26, 95% CI: 0.12-0.58, *p*=0.001) were associated with longer survival (Table 2).

In the adjusted model for patients with LR/DP (n=52), the presence of HPV in the tumour tissue (HR=0.23, 95% CI: 0.62-0.89, *p*=0.034); time to LR/DP >six months (HR=0.20 95% CI: 0.07-0.56, *p*=0.002); high absolute lymphocyte count (HR=0.50, 95% CI: 0.27-0.92, *p*=0.028) and local treatment as a rescue treatment (HR=0.24, 95% CI: 0.07-0.79, *p*=0.019) were independent prognostic factors associated to longer survival (Table 3).

**Figure 3:**
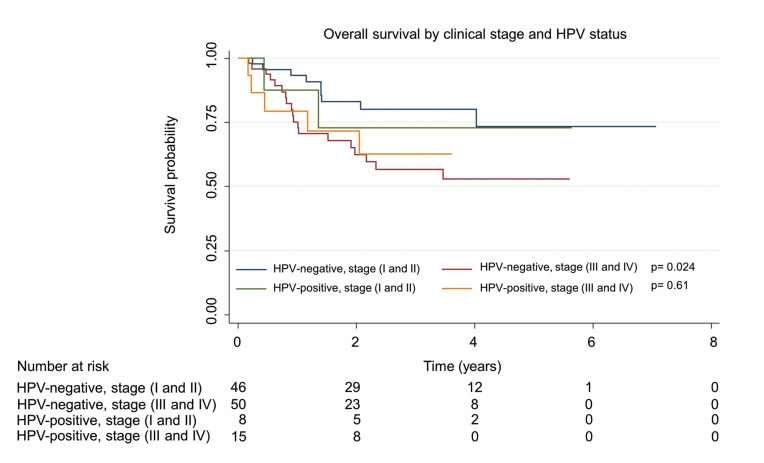
Overall survival by clinical stage illustrated with Kaplan-Meier curve stratified by HPV status. HPV-positive patients had a median of 2.55 years in early stages and 2.05 years in the advanced stage (*p*=0.61), while among patients with HPV-negative tumors, it was 2.37 and 1.90 respectively (*p*=0.024).

Regarding the analysis of the OS by DNA from HPV and by clinical stage, statistically significant differences were found. The median OS in years for patients with HPV-positive tumors was 2.55 in early stages and 2.05 in advanced stages (*p*=0.61) while among patients with HPV-negative tumors, it was 2.37 and 1.90 respectively (*p*=0.024) (Fig. 3).

**Table 2:**
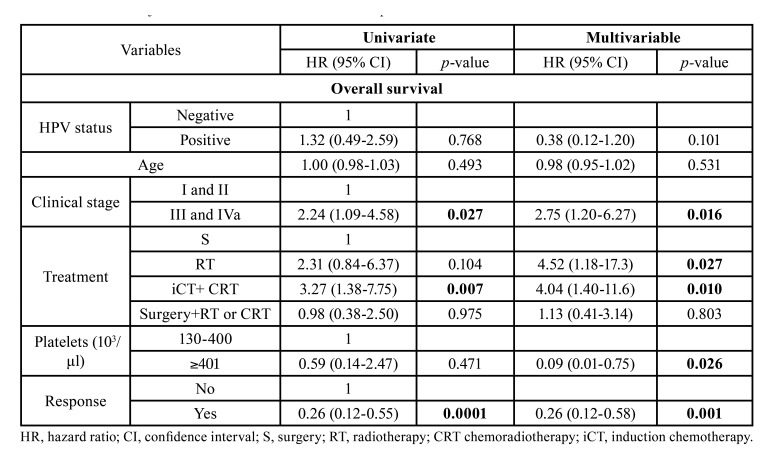
Crude and adjusted HR for overall survival of OSCC patients.

**Table 3:**
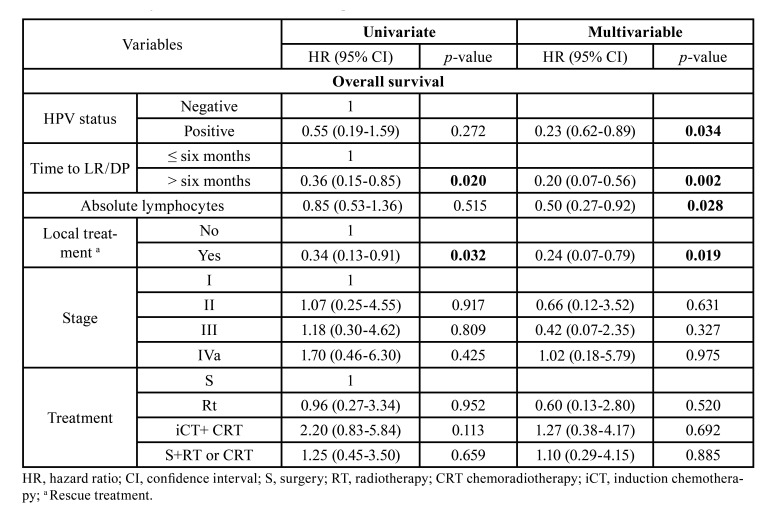
Crude and adjusted HR for overall survival of patients with LR/DP.

## Discussion

Currently, the clinical and prognostic importance of HPV in OSCC is still contradictory, in part due to possible degradation of DNA when detecting HPV DNA from FFPE tissue. Importantly, our analysis showed a significant increased survival of patients with LR/DP who were positive for HPV DNA.

Despite therapeutic efforts, approximately 30-50% of patients with OSCC will never be disease-free or will develop a recurrence (5,6). In a sub-analysis among cases with LR/DP, 52 patients (43.7%) experienced LR/DP with a mean age of 61 years, similar to the 38% reported in a group of patients from Zurich with a mean age of 62 years; likewise, two-thirds of patients with LR/DP occurred in the first two years (15).

Sub-analysis to identify prognostic factors among patients with LR/DP showed four factors associated with more prolonged survival: the presence of HPV in tumor tissue, a higher absolute lymphocyte count before treatment, rescue treatment, and a time to LR/DP > six months after completion of primary treatment. Despite these findings and the increasing diagnosis of HPV-positive associated tumors, the search for HPV is not routinely performed in many settings with limited resources, and the prevalence of HPV in OSCC patients may be underestimated.

Although several factors could influence the estimation of HPV frequency in patients with OSCC (type of specimen and method used), the prevalence of HPV in our cohort (19.3%) is similar to that previously reported in a meta-analysis (11). Detection of HPV-DNA was performed with INNO-LiPA, a test used in epidemiological studies and vaccination trials (16,17), ideal for FFPE samples due to higher sensitivity and capacity to detect HPV even in degraded samples.

Although transcription of HPV genes in OPSCC has been recently studied, there is no consensus on the methodology to be used in the OSCC (18,19). In our study, 22 out of 23 samples that were positive for HPV contained at least one high-risk type (19 with type 16), suggesting its role in tumorigenesis.

Our study showed that HPV status is a prognostic factor for survival of patients with LR/DP; similar to that already reported by OSCC (20) and in the OPSCC (21). We support the conclusion that HPV status is relevant for a favorable prognostic of OSCC patients with LR/DP.

In our study, a higher absolute lymphocyte count was associated with a higher OS in patients with LR/DP in OSCC; although more information is needed, this could reflect a better host-mediated antitumor response. A low absolute lymphocyte count has been associated with poor survival in several types of cancer (22).

Recently it has been described in HPV-negative OPSCC patients that the lymphocyte count could remain low for up to one year, and dysregulation in its count after treatment was associated with worse overall and recurrence-free survival. In patients with OSCC absolute lymphocyte counts and its kinetics in different time points could serve as a useful biomarker for clinical outcomes in patients with OSCC (23).

In the model adjusted for OS, platelet count was an independent factor (*p*=0.010). Values above 400,000 platelets/mm3 were associated with a better prognosis, consistent with a previous report (8). Although there is lacking information on the role of platelets and immunity for HPV infection, pathophysiological mechanisms involving platelet responses have been reported in infections such as HIV, dengue, and influenza pneumonia (24), and this finding warrants further investigation (25). Therefore, our results suggest paying more attention to baseline blood count data, as it may predict the clinical outcome of OSCC patients about OS.

The prognosis for patients not amenable to local rescue treatment with curative intent due to unresecTable disease is very daunting. In our study, the median survival of patients who received palliative treatment (n=37) was only 4.8 months, compared to 11 months in patients who received local rescue treatment. It is well known that patients who are candidates for local rescue therapy have a better prognosis. This suggests that the response to radiotherapy or chemotherapy in OSCC is not as good as that observed in OPSCC. A timely diagnosis of LR/DP may increase the possibility of local rescue treatment (26).

Historically, OSCC has been more frequent in men and has been associated with higher alcohol and tobacco consumption. In this cohort, 57% of patients were women, and only 21% and 30% reported smoking or drinking, respectively. These results are consistent with increasing trend of OSCC among women, as observed in Mexico (27). In this series, nor smoking or alcohol consumption were associated with survival, similar to a study from Portugal (28).

It is particularly relevant to consider whether the presence of HPV could be an important factor explaining younger age in OSCC and not exclusively for OPSCC. In our study, HPV-positive patients were eight years younger than HPV-negative patients (*p*=0.017) consistent, with studies where the average age difference ranges from 4 to 10 years (29).

This study has limitations due to its retrospective nature. The collection of data from clinical records increases the risk of memory and social desirability biases, especially for smoking and alcoholism, with the possible loss of information. Also, some data from clinical records can be lost, not to mention the variability between clinicians and patients´ preferences when choosing treatment.

The median follow-up of patients was 2.17 years, with 51.4% of patients deceased during the first year. In our cohort, as in other studies, the precise date of treatment response was not possible to estimate, but it is indirectly estimated by the date of LR/DP. Also, although we did not determine transcriptional activity, recent information support the claim that HPV is not biologically active in most OSCC cases (18) the presence of DNA from specific types of HPV has prognostic significance in our study.

Despite some limitations, our study has several strengths; i) only included incident cases, ii) dates of LR/DP were corroborated, and iii) presence of DNA from both low and high-risk HPVs was determined. In addition, we identified coinfections with different types HPV, which may provide additional information on the epidemiology of HPV-positive tumors. Future studies should include more cases to determine if identification of HPV types has any value and could include detection of viral activity to assess if it has some role on the prognosis of patients.

## Conclusions

HPV positivity is not associated with longer OS in patients with OSCC, but when LR/DP occurs, patients with HPV-positive tumors have a better prognosis. In these patients, significant factors for increased OS were the presence of HPV in the tumor tissue, a high absolute lymphocyte count, time to LR/DP > six months, and local treatment with rescue therapy. Identification of these prognostic factors may improve clinical surveillance and treatment of patients with LR/DP.
